# Social interactions and affective neuroscience personality traits among Chinese educators: a randomized intervention study on wellbeing

**DOI:** 10.3389/fpsyg.2026.1712521

**Published:** 2026-02-20

**Authors:** Chao Li, Ying Li

**Affiliations:** Department of Education, Yunnan Normal University, Kunming, Yunnan, China

**Keywords:** Affective Neuroscience Theory, ANPs, communication training, emotional regulation, emotional traits, intervention study, social satisfaction, stress management

## Abstract

**Background:**

Teacher wellbeing is increasingly recognized as a key determinant of instructional quality and professional sustainability. However, evidence-based interventions grounded in neurobiological models of emotion remain limited in educational contexts. Drawing on Affective Neuroscience Theory (ANT), which conceptualizes primary emotional systems such as SEEKING, CARING, and PLAYING as foundational to adaptive functioning, this study examines whether brief, well-structured interventions can modulate trait-level emotional functioning and relate to occupational and social outcomes among teachers. Here, we focus on ANT-defined trait-like primary emotional systems rather than broader applied constructs (e.g., emotional intelligence), and we interpret changes as shifts in trait-level affective functioning.

**Methods:**

A randomized controlled trial was conducted with 182 Chinese secondary school teachers assigned to a four-week social interaction stress management program, a four-week communication skills training program, or a no-intervention control group. The two interventions followed a parallel structure but targeted distinct affective pathways. Primary emotional traits were assessed using the Chinese Affective Neuroscience Personality Scales, and work engagement and satisfaction with social interactions were measured with validated self-report instruments. Mixed-design ANOVAs were used to evaluate changes from pre-intervention to post-intervention and to a three-month follow-up.

**Results:**

Compared with the control group, both intervention groups showed greater increases in positive primary emotional systems (SEEKING, CARING, and PLAYING) and reductions in negative affective systems (FEAR and SADNESS, and to a lesser extent ANGER), with changes generally more pronounced in the stress management condition. Modest improvements in work engagement and satisfaction with social interactions were also observed in the intervention groups. Most emotional and occupational changes were maintained at the three-month follow-up.

**Discussion:**

The findings suggest that brief, theory-driven interventions may be associated with meaningful changes in teachers’ affective profiles and related occupational outcomes. By applying an ANT-based framework within an educational setting, this study contributes to the growing literature on affective plasticity and teacher wellbeing and highlights the potential value of neuroscience-informed approaches in professional development. Future research should further examine underlying mechanisms, longer-term sustainability, and cross-cultural generalizability. Conceptually, the study helps position ANT-based primary emotional systems as modifiable affective dispositions within teacher professional development, offering an experimentally grounded complement to the broader teacher wellbeing intervention literature.

## Introduction

In recent years, there has been growing interest in how affective processes shape occupational wellbeing and professional performance (Kelloway et al., 2023). This is particularly relevant in educational and caregiving settings, where emotional competence plays a crucial role in shaping interpersonal dynamics and fostering long-term resilience (Beames et al., 2023). Grounded in Jaak Panksepp’s Affective Neuroscience Theory (ANT) (Panksepp, 1982, 2004), this study examines the impact of targeted interventions—specifically, stress management and communication skills training—on core emotional dispositions, work engagement, and satisfaction with social interaction (Cook et al., 2020). Despite increased recognition of the importance of emotional functioning in teachers, few empirical studies have implemented ANT-based interventions in educational contexts, and even fewer have examined whether brief, theory-driven programs can be associated with changes in trait-level affective systems rather than surface-level skills or states, leaving a significant gap regarding whether primary emotional systems can be modified through structured professional development programs.

The Affective Neuroscience Theory proposes the existence of evolutionarily conserved primary emotional systems that are anchored in subcortical structures of the mammalian brain. These include positive systems—SEEKING, CARING, and PLAYING—and negative systems—FEAR, ANGER, and SADNESS (Panksepp, 2005). Each of these systems contributes to the regulation of behavior, affective style, and personality traits in a bottom-up fashion, from affective circuits to higher-order cognition and behavior (Montag and Davis, 2018). Importantly, individual differences in the reactivity or sensitivity of these systems can be quantified using instruments such as the Affective Neuroscience Personality Scales (ANPS), providing a biologically grounded framework for investigating trait emotionality (Panksepp, 2011). In the present study, “emotional states” refer to the activation of these primary systems, whereas “emotional resilience” describes the capacity to modulate such activation adaptively across time. “Emotional intelligence,” in contrast, reflects a broader set of cognitive–behavioral competencies and is not interchangeable with ANT-based constructs. These distinctions are central to the present work and inform the conceptual assumptions guiding the interpretation of the intervention effects.

Research increasingly supports the idea that targeted interventions can produce measurable affective benefits even when delivered over short time frames. Several recent randomized trials and meta-analyses show that brief cognitive reappraisal trainings, ultra-short mindfulness programs, and concise positive psychology interventions can yield significant changes in emotional regulation, affective experience, and wellbeing (Barcaccia et al., 2024; Morello et al., 2023; Rubin et al., 2024; Wolfe et al., 2023; Zhu et al., 2025; Kirca et al., 2023; Predatu et al., 2024). These studies collectively demonstrate that affective plasticity can be activated within tightly structured interventions, challenging the notion that meaningful affective change necessarily requires long or intensive programs, and providing empirical justification for the four-week program format used in this work.

Building on this theoretical foundation, the current experimental study applied a mixed design to assess the impact of two psychological training interventions—stress management and effective communication—on the modulation of primary emotional traits, as well as their downstream influence on work engagement and satisfaction with social interactions. The programs targeted distinct affective pathways: the stress management intervention emphasized downregulation of negative systems (FEAR, SADNESS, ANGER), whereas the communication skills program emphasized activation of positive systems (CARING, PLAYING, SEEKING) through interpersonal skill development. By comparing intervention groups with a non-treatment control, we aimed to determine whether targeted training can reshape trait-level affective profiles and enhance psychosocial functioning in the workplace. Rather than testing specific causal mechanisms, the study adopts a theory-driven approach to examine patterns of affective change consistent with ANT assumptions. The longitudinal design enabled follow-up assessments to examine whether the observed changes were sustained over time, thereby adding a developmental dimension to the findings.

This investigation thus integrates biologically grounded models of personality with applied psychological intervention, filling a critical gap in the literature that often treats personality traits as static rather than malleable constructs. By focusing on the modulation of core affective systems rather than surface-level behaviors, the study contributes to a deeper understanding of the mechanisms through which personal development and professional performance can be enhanced. The primary aim of this study was to evaluate the effectiveness of brief psychological interventions in modulating primary emotional systems and improving social and occupational outcomes. In doing so, it extends prior research on teacher wellbeing by applying an affective neuroscience framework to an educational intervention context, and identifies potential pathways through which emotional interventions may promote teacher wellbeing.

## Literature review

### Modulating primary emotional traits through intervention

Affective Neuroscience Theory (ANT), developed by Jaak Panksepp, identifies primary emotional systems that are evolutionarily conserved, subcortically anchored, and crucial to shaping personality and behavior. These systems include SEEKING, CARING, PLAYING, FEAR, ANGER, and SADNESS, and they can be empirically measured using instruments like the Affective Neuroscience Personality Scales (ANPS) (Davis and Panksepp, 2018; Montag et al., 2021). Individual differences in these systems influence emotional reactivity, resilience, and motivational drive. Although these systems have trait-like properties, ANT conceptualizes them as dynamic affective dispositions whose activation can shift in response to structured environmental or experiential input (Panksepp, 2010; Panksepp et al., 2014).

Recent evidence suggests that targeted psychological interventions, such as stress reduction techniques or social skills training, can lead to measurable changes in these emotional systems. For example, SEEKING (curiosity, motivation) and PLAYING (joyful social engagement) are particularly responsive to enriched interpersonal and learning environments (Garland et al., 2010; Panksepp, 2017). Conversely, FEAR and SADNESS systems—often implicated in stress vulnerability—can be downregulated through affect-focused resilience programs (Dolcos et al., 2021). Interventions that enhance emotional regulation also activate neural pathways tied to safety, reward, and reduced threat reactivity (Ochsner and Gross, 2008).

Importantly, a growing body of empirical research demonstrates that even brief, tightly structured interventions can produce meaningful affective improvements. Randomized trials involving single-session or ultra-brief cognitive reappraisal training have shown immediate reductions in anxiety and stress-related responses (Wolfe et al., 2023), while short-format mindfulness programs delivered over one to four sessions have produced measurable gains in emotional wellbeing (Barcaccia et al., 2024; Rubin et al., 2024). Meta-analytic evidence also indicates that concise mHealth-based reappraisal interventions can reliably enhance emotion regulation (Morello et al., 2023), and short positive psychology protocols such as gratitude-based exercises have been found to increase positive affect and wellbeing (Kirca et al., 2023; Predatu et al., 2024). Workplace studies similarly show that brief reappraisal-based interventions can influence emotional experience and performance within occupational settings (Zhu et al., 2025). Together, these findings support the notion of affective plasticity and provide a strong empirical rationale for employing a four-week training design in the present research.

Despite these advances, the application of ANT-based interventions in educational occupational contexts remains limited. Existing work has largely focused on clinical populations or general adult samples, leaving open the question of whether primary emotional systems can be modulated in teachers, a group facing unique and sustained affective demands. Considering the documented associations between teachers’ emotional functioning, interpersonal dynamics, and occupational wellbeing (Beames et al., 2023), examining whether structured interventions can influence ANT-defined emotional systems in this population represents a significant gap in the literature.

These findings support Hypothesis 1, which posits that brief, well-structured interventions can enhance positive primary emotions (SEEKING, CARING, PLAYING) and reduce negative emotions (FEAR, ANGER, SADNESS), demonstrating emotional trait plasticity even over short time scales. Hence, we expected that the Stress Management and Communication Skills group participants would show greater improvements in positive emotional states (SEEKING, CARING, PLAYING) and reductions in negative states (FEAR, ANGER, SADNESS) compared to the control group. This assumption is grounded in ANT’s premise that emotionally salient learning experiences can influence subcortical affective circuits, and it is further supported by empirical demonstrations of rapid emotional change following short-format psychological interventions (Barcaccia et al., 2024; Wolfe et al., 2023; Zhu et al., 2025).

### Emotional traits, social interactions, and work engagement

Emotional tendencies, especially those measured through ANT, are deeply interwoven with workplace behaviors and interpersonal satisfaction. The SEEKING system fuels exploration and cognitive engagement, which translates into higher motivation and work enthusiasm, while CARING facilitates empathy and supportive social interactions (Lu and Jian, 2024). These systems correlate strongly with constructs like work engagement, which encompasses vigor, dedication, and absorption (Diener et al., 2020). PLAYING, which reflects social joy and flexible interactional patterns, has also been linked to prosocial behavior and the maintenance of positive social exchanges—factors that support the formation of cohesive professional relationships.

Work environments characterized by emotional warmth, collaboration, and psychological safety are more likely to activate positive emotion systems, fostering individual wellbeing and group cohesion. Conversely, negative systems like FEAR and ANGER are associated with burnout, withdrawal, and social friction (Davidson et al., 2000). Interventions that reduce stress or enhance communication appear especially effective in this regard. Programs promoting emotional self-regulation and conflict resolution directly impact the affective underpinnings of social behavior, thereby enhancing satisfaction in interpersonal exchanges and fostering workplace engagement over time (Montag et al., 2021). Recent research has further shown that brief trainings aimed at regulating emotional responses or improving interpersonal awareness can produce measurable gains in social connectedness and occupational functioning, suggesting that emotional traits play a mediating role between intervention content and workplace outcomes (Beames et al., 2023).

Thus, the literature supports Hypothesis 2: enhancing positive emotional systems through targeted interventions is likely to improve satisfaction with social interactions and levels of work engagement. Hence, we expected that the participants in the experimental groups would report higher satisfaction with social interactions and work engagement levels post-intervention than the control group. This assumption is grounded in evidence linking activation of SEEKING, CARING, and PLAYING to motivational and relational outcomes, as well as in empirical demonstrations that changes in emotional functioning can generalize to broader workplace behaviors when interventions target the affective mechanisms underlying social engagement.

### Long-term maintenance of emotional and occupational outcomes

A critical question in intervention research is whether gains in emotional regulation and engagement are sustainable (Dreer and Gouasé, 2022). From the perspective of ANT, emotional traits are deeply rooted but not fixed; neuroplasticity enables these systems to adapt in response to persistent environmental or experiential influences (Davidson et al., 2000). Recent work on short-format emotion regulation and mindfulness training indicates that such adaptations can emerge even after brief interventions, provided that individuals continue to activate and rehearse the strategies introduced during training (Wolfe et al., 2023). Sustained changes in emotional tendencies—particularly increases in SEEKING and decreases in FEAR/SADNESS—are achievable when interventions are consistently reinforced or align with the individual’s motivational architecture (Craske et al., 2016).

Evidence from both clinical and organizational contexts confirms that maintaining positive affect is linked to better long-term outcomes in mental health, workplace engagement, and interpersonal functioning (Quoidbach et al., 2015). In organizational settings, even relatively small affective shifts have been shown to accumulate over time, influencing job satisfaction and interpersonal functioning in lasting ways. Interventions that combine emotional awareness with behavioral strategies show the most durable effects, especially when integrated into daily routines or organizational culture (Dolcos et al., 2021).

**Figure 1:**
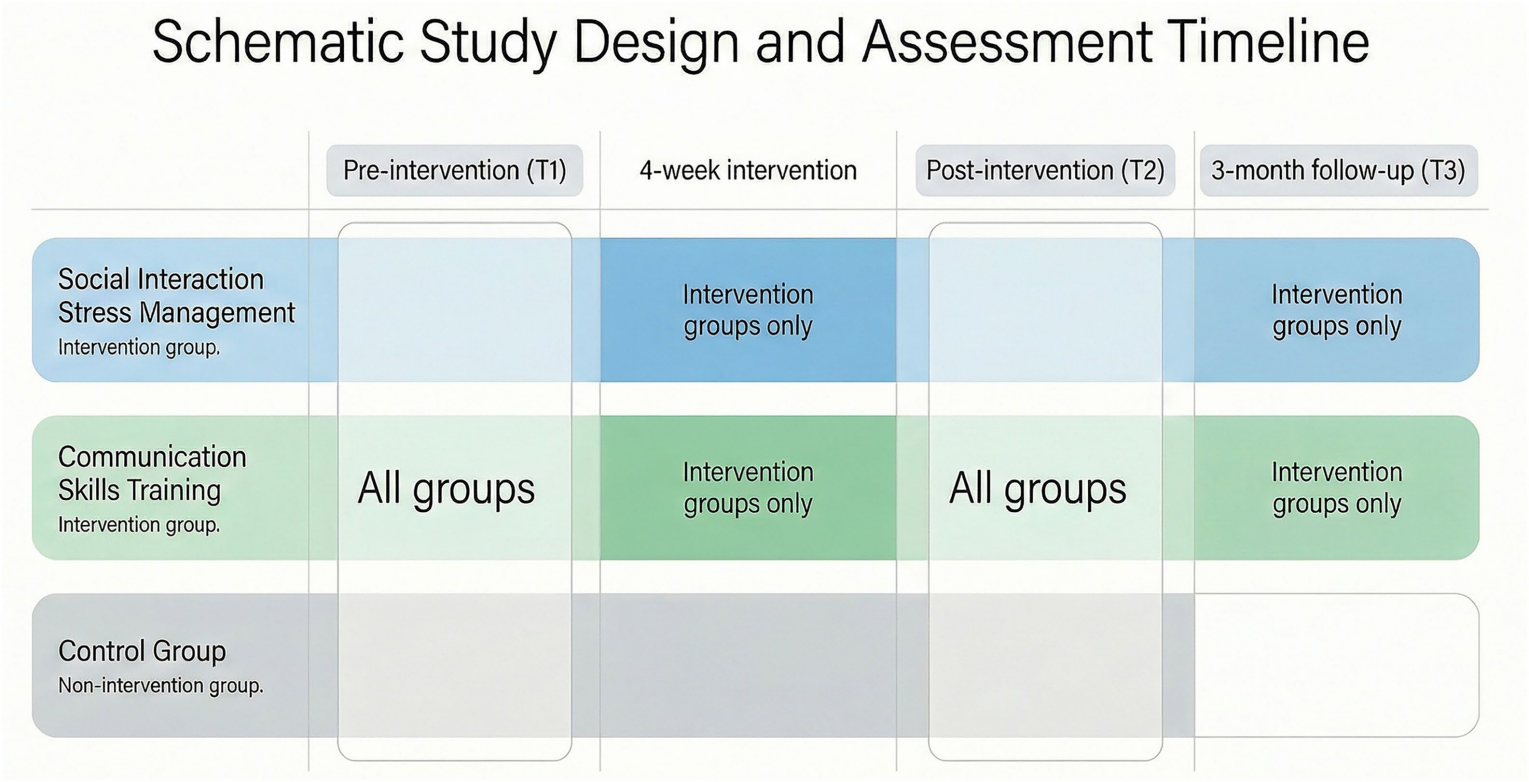
Schematic representation of the randomized study design and assessment timeline. The figure illustrates the temporal structure of the randomized controlled trial, including pre-intervention assessment (T1), a four-week intervention period for the two experimental groups, post-intervention assessment (T2), and a three-month follow-up assessment (T3) conducted only for the intervention groups.

Therefore, available research substantiates Hypothesis 3: Improvements in emotional states, interpersonal satisfaction, and work engagement can be maintained over extended periods, particularly when interventions target affective systems at their neuropsychological roots. For this reason, the present study anticipated that emotional and occupational gains would remain evident at the end of the academic year, reflecting a consolidation process commonly described in longitudinal affective intervention research. Hence, we expected the improvements in emotions, satisfaction with social interactions, and Work engagement observed post-intervention would be maintained at the end of the academic year (Figure 1).

## Method

### Participants

A total of 200 secondary school teachers were assessed for eligibility. Thirteen individuals were excluded—seven for not meeting the inclusion criteria and six who declined to participate—resulting in 187 eligible participants who were randomly assigned to one of three groups. Five participants did not complete the baseline assessment and were therefore excluded from the analyses, yielding a final sample of 182 teachers. Participants were allocated to a control group (*n* = 62), a social interaction stress management group (*n* = 58), and an effective communication skills training group (*n* = 62).

Randomization was conducted using a computer-generated allocation sequence to ensure equal assignment probability across groups. Before the intervention phase, we examined whether the randomization procedure produced equivalent groups at baseline. One-way ANOVAs showed no significant differences among the three groups in age, *F*(2, 179) = 1.93, *p* = 0.148, and gender, *F*(2, 179) = 1.57, *p* = 0.211. These results confirmed that the allocation process yielded comparable groups prior to the intervention.

### Procedure

Ethical approval for the study was granted by the Ethics Committee of Yunnan Normal University (ID: EDU-386542-04) on 27 November 2024. All procedures complied with institutional guidelines and the Declaration of Helsinki. Recruitment took place during December 2024 and January 2025 through announcements and posters distributed across secondary schools in several districts of Yunnan province. Interested teachers contacted the research team and received detailed information about the study. Electronic informed consent was obtained immediately before completing the baseline assessment, which was administered at the end of January 2025 through a secure online platform.

Participants were then randomly assigned to one of three conditions: a control group, a communication skills training program, or a stress management intervention focused on social interactions. Randomization was carried out using a computer-generated sequence to ensure equal assignment probability across groups. Baseline questionnaires were completed online by all participants who consented and met the eligibility criteria.

The two intervention programs were delivered in person by one trained facilitator from the research team. Both programs were implemented over four consecutive weeks, with one session held per week. To avoid disruptions associated with the Chinese New Year holiday period, the intervention phase was scheduled between early March and early April 2025. Post-intervention assessments were completed online immediately after the final session in April. In summary, baseline assessment took place in late January 2025, the four-session intervention ran from early March to early April 2025, post-test was completed immediately after the final session in April, and follow-up was conducted online in July 2025 (≈3 months’ post-test).

Only participants in the two intervention groups were invited to complete the follow-up assessment, as long-term maintenance of intervention effects was relevant exclusively for individuals who received active training. The follow-up evaluation was conducted online in July 2025, approximately 3 months after the post-test, coinciding with the end of the academic term.

**Figure 2:**
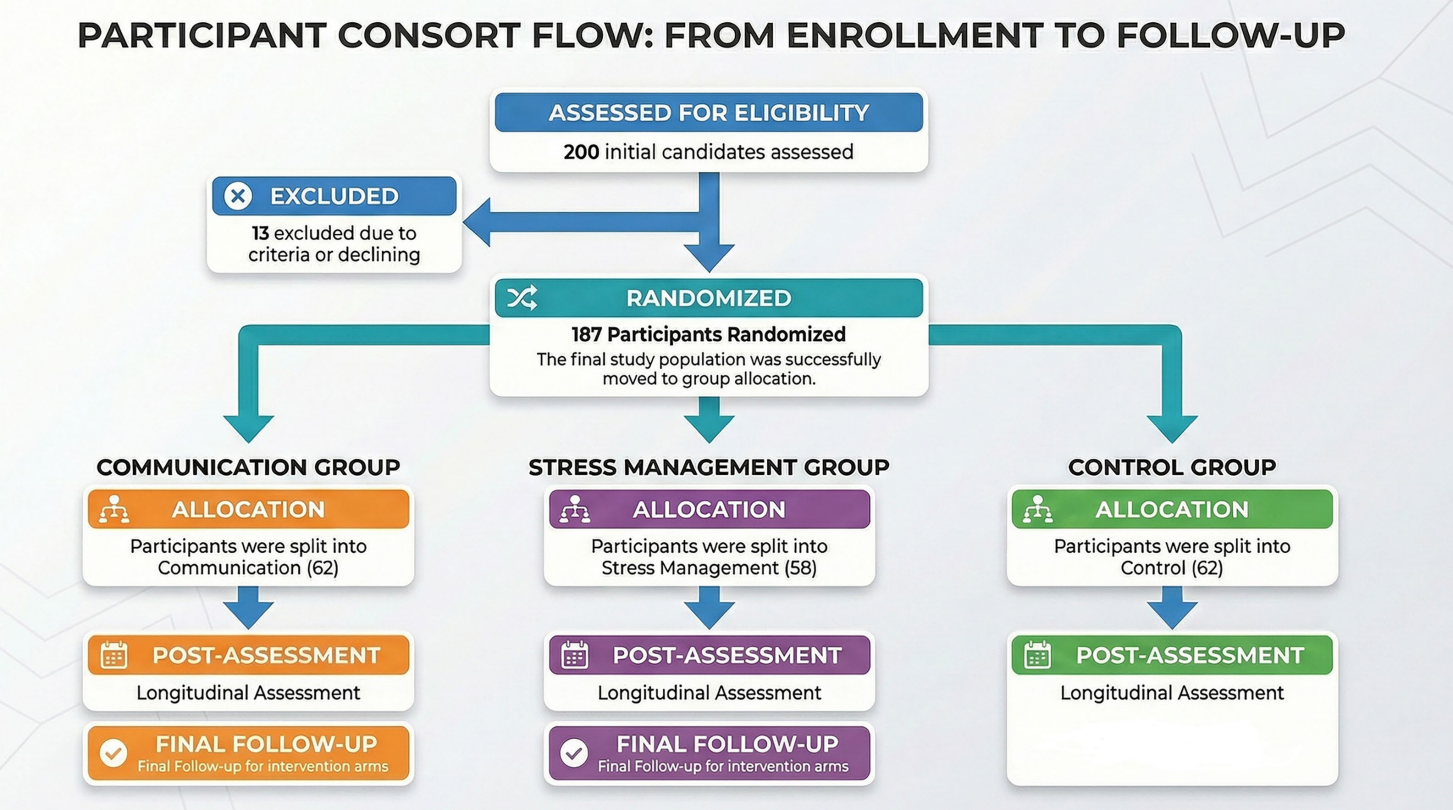
CONSORT diagram.

Attendance requirements were applied to ensure compliance with the intervention protocols. Two participants in the communication group and four participants in the stress management group were excluded for attending fewer than 80% of the sessions. All participants in the control group completed the post-test and were retained for analysis. In total, data from 182 teachers were included in the pre- and post-intervention analyses, and follow-up data were obtained for the two intervention groups. A detailed overview of participant flow is presented in Figure 2 (CONSORT diagram).

It should be noted that the assumptions referred to throughout the manuscript are conceptual and theory-driven in nature rather than statistical assumptions. These assumptions reflect the theoretical framework of Affective Neuroscience Theory and guide the interpretation of the intervention effects, but they do not impose additional constraints on the statistical analyses conducted in this study.

### Interventions

The four-week format was selected because evidence from brief, tightly structured interventions indicates that measurable affective and regulatory changes can emerge over short time frames when sessions are standardized and practice-based. Two active intervention programs were implemented: a Communication Skills Training program and a Social Interaction Stress Management program. Both interventions were designed as brief, structured, group-based trainings delivered over four consecutive weeks, with one session per week lasting approximately 2 h. The programs shared a common pedagogical framework and delivery format but differed in their primary affective targets and theoretical emphasis.

### General intervention framework and relationship between programs

The two programs were conceived as complementary but theoretically distinct approaches to improving teachers’ emotional functioning and wellbeing. Both were grounded in Affective Neuroscience Theory (ANT), which posits that primary emotional systems represent biologically rooted affective dispositions that can be modulated through emotionally salient learning experiences. Within this framework, the interventions were designed to influence emotional functioning via different but convergent affective pathways.

Specifically, the Social Interaction Stress Management program primarily targeted the downregulation of negative primary emotional systems (FEAR, SADNESS, and ANGER) by reducing stress reactivity and enhancing emotional regulation in socially demanding situations. In contrast, the Communication Skills Training program primarily focused on activating and strengthening positive primary emotional systems (SEEKING, CARING, and PLAYING) through the development of interpersonal skills, empathic communication, and positive social engagement.

Despite these different emphases, both programs were aligned in structure, duration, group format, and level of facilitator guidance. This parallel design allowed for meaningful comparison between interventions while ensuring that any observed differences could be interpreted in terms of theoretical orientation and affective targets, rather than procedural inconsistencies.

### Design rationale and content of the communication skills training program

The Communication Skills Training program was designed to enhance participants’ competence in verbal and nonverbal communication, empathic understanding, and active listening during everyday social interactions. From an ANT perspective, these skills are closely linked to the activation of CARING (empathy and nurturing tendencies), PLAYING (social joy, flexibility, and positive interaction), and SEEKING (curiosity and engagement with others).

The program followed principles of experiential learning and interpersonal skills training. Activities included brief psychoeducational inputs, structured role-playing exercises, guided group discussions, and feedback-based reflection. Each session targeted a specific set of interpersonal competencies, progressing from foundational awareness of communication processes to the integration and application of skills in realistic social scenarios. The repeated use of role-play and group interaction was intended to create emotionally salient learning contexts that could stimulate positive affective systems through practice, feedback, and social reinforcement.

### Design rationale and content of the social interaction stress management program

The Social Interaction Stress Management program aimed to equip participants with strategies to manage emotional reactivity and interpersonal stress. This intervention was theoretically oriented toward reducing chronic activation of FEAR, SADNESS, and ANGER, which are often associated with social threat, frustration, and emotional overload in occupational contexts.

Drawing on mindfulness-based and cognitive-behavioral principles, the program combined psychoeducation on stress processes with practical regulation techniques. Core activities included mindfulness and attention regulation exercises, breathing techniques, cognitive restructuring of stress-related social appraisals, and the development of individualized coping plans. These components were selected to enhance emotional awareness, increase perceived control in social situations, and reduce automatic stress responses, thereby facilitating adaptive modulation of negative affective systems.

The emphasis on repeated practice and real-life application was intended to promote the internalization of regulation strategies, allowing participants to apply them beyond the training context and across diverse social interactions in their professional and personal lives.

Ensuring suitability, coherence, and fidelity of the interventions: several steps were taken to ensure that the activities within each program were suitable for their intended affective targets and coherently aligned with the overall intervention rationale. First, all sessions were delivered by a trained facilitator using standardized session plans developed by the research team. This ensured consistency in content delivery and adherence to the intended theoretical framework.

Second, both programs followed a parallel session structure—including psychoeducation, experiential exercises, and group reflection—allowing participants to engage actively with the material while maintaining comparability across conditions. Third, the content of each session was designed to progressively build on previously introduced concepts and skills, supporting cumulative learning and emotional engagement over time.

Finally, a manipulation check was administered immediately after the intervention to verify participants’ engagement with, and understanding of, the core components of each program. The results of this check supported the fidelity of implementation and confirmed that participants had been exposed to and understood the intended intervention content.

### Instruments

#### Measurement of emotions

Primary emotional traits were assessed with the Chinese version of the Affective Neuroscience Personality Scales (ANPS), validated for use in Chinese-speaking populations (Sindermann et al., 2018; Lu and Jian, 2024). The ANPS is grounded in Panksepp’s affective neuroscience framework, which conceptualizes individual differences in emotional functioning as reflecting the activity of evolutionarily conserved primary emotional systems embedded in subcortical brain structures. These systems represent biologically based affective dispositions rather than transient emotional states.

Six primary emotional systems were measured, each assessed with six items rated on a 5-point Likert scale ranging from 1 (strongly disagree) to 5 (strongly agree). The positive affect dimensions included CARING (empathy, nurturing tendencies, prosocial concern), SEEKING (curiosity, exploratory drive, goal-oriented behavior), and PLAYING (social joy, humor, light-hearted interaction). The negative affect dimensions included ANGER (frustration, irritability, proneness to aggression), FEAR (anxiety, threat sensitivity, avoidance tendencies), and SADNESS (separation distress, grief, social withdrawal). Higher scores reflect stronger activation of the corresponding emotional system. Internal consistency in the present sample was excellent for ANGER (*α* = 0.91, *ω* = 0.91), SADNESS (*α* = 0.91, *ω* = 0.91), FEAR (*α* = 0.93, *ω* = 0.93), SEEKING (*α* = 0.92, *ω* = 0.92), and PLAYING (*α* = 0.93, *ω* = 0.93), and good for CARING (*α* = 0.87, *ω* = 0.87). These coefficients indicate that all six emotional systems were measured with high reliability.

#### Measurement of work engagement

Work engagement was assessed with the Chinese version of the Utrecht Work Engagement Scale–Short Form (C-UWES-3), an ultra-brief adaptation validated for use in Chinese occupational settings (Lam et al., 2022). The UWES conceptualizes engagement as a positive, fulfilling, work-related state characterized by vigor, dedication, and absorption. The C-UWES-3 includes three items, one representing each dimension, rated on a 5-point Likert scale from 1 (never) to 5 (always/every day). Despite its brevity, the scale has shown sound psychometric properties and coherent associations with constructs central to the Job Demands–Resources model in Chinese samples. Internal consistency for the three-item engagement index was good (*α* = 0.84, *ω* = 0.84), supporting its reliability as a concise indicator of professional engagement suitable for repeated measurement.

#### Measurement of social interaction satisfaction

Social interaction satisfaction was measured using a multidomain index adapted from validated instruments used in large-scale studies of social wellbeing (Bai et al., 2019; Xu et al., 2023). Participants rated their satisfaction across five relational domains: relationships with a partner or closest person; family relationships; friendships; work-, career-, or study-related social connections; and participation in social and leisure activities. Each domain was assessed with the question: “In general, how satisfied are you with what you have achieved in your life so far in the area of [domain]?”, rated from 1 (very dissatisfied) to 5 (very satisfied). Domain scores were averaged to create an overall satisfaction index. Internal consistency for this measure in the present sample was good (*α* = 0.86, *ω* = 0.86), indicating reliable assessment of perceived social fulfillment across relational contexts.

#### Manipulation check

A manipulation check was administered immediately after the final intervention session to verify participants’ engagement with, and understanding of, the program content. In the Communication Skills Program, participants responded to items assessing comprehension of verbal and nonverbal communication strategies, the role of active listening, and the application of empathy in social interactions, along with recall of role-play activities and feedback received during group discussions. In the Stress Management Program, items assessed knowledge and use of core techniques introduced during the sessions, including mindfulness practices, basic cognitive-behavioral strategies for managing social stress, and peer support exercises, with additional items probing the application of these techniques in daily life. The manipulation check served to confirm fidelity to the intervention protocols and to support the internal validity of subsequent analyses.

#### Data analyses

Data analyses proceeded in several steps to evaluate the effectiveness of the interventions and to examine the relationships among emotional states, social interaction satisfaction, and work engagement. First, descriptive statistics and Pearson correlations were computed for all primary variables, including the six emotional traits from the Affective Neuroscience Personality Scales (ANPS), satisfaction with social interactions, and work engagement scores at each wave.

To evaluate the immediate effects of the interventions on primary emotions and outcome variables (Hypotheses 1 and 2), a series of mixed-design (repeated-measures) ANOVAs were conducted with Time (pre-intervention vs. post-intervention) as a within-subjects factor and Group (control, communication skills, and stress management) as a between-subjects factor. These analyses tested main effects of Time and Group, as well as Time × Group interaction effects for each dependent variable (SEEKING, CARING, PLAYING, FEAR, ANGER, SADNESS, work engagement, and social interaction satisfaction).

To examine the maintenance of changes at follow-up (Hypothesis 3), repeated-measures ANOVAs with three time points (pre-intervention, post-intervention, and follow-up at the end of the academic year) were conducted including only the two intervention groups (communication skills and stress management). For each outcome, Time was specified as a within-subjects factor and Group as a between-subjects factor.

For all repeated-measures analyses, Mauchly’s test of sphericity was inspected. When the sphericity assumption was violated, Greenhouse–Geisser corrections were applied to the degrees of freedom. Where omnibus tests were statistically significant, follow-up contrasts and pairwise comparisons with Bonferroni adjustment were used to clarify the pattern of change over time and differences between groups. All analyses were performed using IBM SPSS Statistics, Version 29.01, and effect sizes (partial eta squared, *η*^2^ₚ) were reported alongside *p*-values to aid interpretation of the practical significance of the findings (Table 1).

**Table 1:** Demographic characteristics of participants by group.

Variable	Control (*n* = 62)	Stress management (*n* = 58)	Communication skills (*n* = 62)	Total (*N* = 182)
Age range	25–47	25–49	25–49	25–49
Most frequent age	29	Early 30s	30	—
% Women	62.9%	50%	51.7%	—
Marital status	58.1% married/partnered 6.5% divorced 35.5% single	40.3% married/partnered 12.9% divorced 40.3% single 6.5% widowed	56.9% married/partnered 5.2% divorced 37.9% single	—

Values reflect self-reported demographic characteristics provided at baseline. No significant between-group differences were detected.

## Results

### Descriptive and correlational statistics

Table 2 presents the means, standard deviations, and sample sizes for all primary study variables, including trait-level positive and negative emotions, work engagement, and satisfaction with social interactions across three time points: pre-intervention, post-intervention, and three-month follow-up. Descriptive statistics indicate moderate levels of positive affect at baseline, with average scores of *M* = 2.46 (SEEKING), *M* = 3.60 (CARING), and *M* = 3.23 (PLAYING). Post-intervention, increases were observed across these dimensions, especially in CARING (*M* = 4.04) and PLAYING (*M* = 3.49). Reductions were noted in negative affect scores, particularly SADNESS (*M* = 3.45–3.22) and FEAR (*M* = 3.30–3.13). Similar trends were observed for work engagement and satisfaction with social interaction over time.

**Table 2:** Descriptive statistics for the study variables.

Variable	*M*	SD	*N*
1. Seeking	2.4577	0.53537	182
2. Caring	3.5962	0.4385	182
3. Playing	3.2287	0.62505	182
4. Seeking Post	2.7187	0.61759	182
5. Caring Post	4.0396	0.58619	182
6. Playing Post	3.4945	0.48614	182
7. Seeking End Course	2.7750	0.6552	120
8. Caring End Course	3.9950	0.56537	120
9. Playing End Course	3.4448	0.55873	120
10. Anger	3.4066	0.61354	182
11. Sadness	3.4469	0.54558	182
12. Fear	3.2985	0.73541	182
13. Anger Post	3.3901	0.52308	182
14. Sadness Post	3.5751	0.58225	182
15. Fear Post	3.1337	0.78025	182
16. Anger End Course	3.1972	0.4563	120
17. Sadness End Course	3.2241	0.49103	116
18. Fear End Course	3.1056	0.85119	120
19. Work engagement	2.9912	0.50273	182
20. Work engagement Post	3.0253	0.46918	182
21. Work engagement End Course	3.0867	0.40396	120
22. Satisfaction Social Interaction	2.6494	0.42275	180
23. Satisfaction Social Interaction Post	2.6793	0.49283	179
24. Satisfaction Social Interaction End Course	2.6438	0.49261	112

Values represent mean scores per item. All scales were rated on a 1–5 scale.

Bivariate correlations were largely consistent with expectations (see Supplementary Table S1). Positive primary emotional systems showed positive intercorrelations and tended to relate positively to work engagement and social interaction satisfaction, whereas negative systems showed the opposite pattern. For example, the FEAR post was negatively associated with the SEEKING post (*r* = −0.36, *p* < 0.001) and with the Social Satisfaction post (*r* = −0.57, *p* < 0.001).

### Hypotheses testing

To ensure a coherent presentation aligned with Affective Neuroscience Theory, all ANOVAs are reported following the same ordered sequence of primary emotional systems: SEEKING, CARING, and PLAYING, followed by FEAR, ANGER, and SADNESS.

*Hypothesis 1:* Experimental groups will show greater improvements in positive emotional states (SEEKING, CARING, PLAYING) and reductions in negative states (FEAR, ANGER, SADNESS) compared to the control group.

To test whether participants in the stress management and communication skills groups showed greater improvements in positive emotional states compared to the control group, three mixed-design ANOVAs were conducted with Time (pre-intervention and post-intervention) as a within-subjects factor and Group (control, stress management, communication) as a between-subjects factor. The dependent variables were SEEKING, CARING, and PLAYING, as Table 3 shows.

**Table 3:** Estimated marginal means for positive emotions by group and time.

Emotion	Group	Time	Mean	SE	95% CI Lower	95% CI Upper
SEEKING	Control	Pre	2.511	0.074	2.365	2.657
		Post	2.555	0.072	2.413	2.697
	Stress Mgmt	Pre	2.461	0.065	2.332	2.591
		Post	2.832	0.081	2.672	2.992
	Communication	Pre	2.397	0.067	2.264	2.531
		Post	2.772	0.081	2.612	2.932
CARING	Control	Pre	3.492	0.061	3.370	3.613
		Post	3.768	0.078	3.614	3.922
	Stress Mgmt	Pre	3.681	0.053	3.577	3.785
		Post	4.313	0.062	4.191	4.434
	Communication	Pre	3.617	0.054	3.511	3.722
		Post	4.038	0.068	3.904	4.171
PLAYING	Control	Pre	3.250	0.071	3.109	3.391
		Post	3.234	0.075	3.085	3.383
	Stress Mgmt	Pre	3.286	0.076	3.135	3.438
		Post	3.784	0.042	3.701	3.867
	Communication	Pre	3.144	0.094	2.958	3.331
		Post	3.463	0.043	3.378	3.549

SE, Standard Error; CI, Confidence Interval.

Mauchly’s Test of Sphericity indicated that the assumption of sphericity was met for all three positive emotion variables, as the test statistic was 1.000 with no significant violations observed. Therefore, unadjusted degrees of freedom were used in interpreting the within-subject effects. The analysis revealed significant main effects of Time across all three positive emotions, indicating that positive affect increased from pre- to post-intervention regardless of group. In addition, significant time-by-group interaction effects were found for all variables, indicating that the degree of improvement in positive emotional states varied across groups.

As Table 4 shows, estimated marginal means indicated that while all groups showed some degree of improvement in SEEKING, CARING, and PLAYING over time, the social interaction stress management and effective communication groups exhibited greater gains compared to the control group. *Post hoc* comparisons confirmed statistically significant differences between the experimental groups and the control group for CARING and PLAYING, particularly favoring the stress management condition.

**Table 4:** Multivariate and univariate ANOVA results for positive emotions.

Emotion	Effect	*F*	df (Effect)	df (Error)	*p*-value	Partial *η*^2^
SEEKING	Time	28.84	1	179	<0.001	0.139
	Time × Group	5.08	2	179	0.007	0.054
	Group	0.91	2	179	0.403	0.010
CARING	Time	137.25	1	179	<0.001	0.434
	Time × Group	7.66	2	179	<0.001	0.079
	Group	11.81	2	179	<0.001	0.117
PLAYING	Time	44.59	1	179	<0.001	0.199
	Time × Group	14.52	2	179	<0.001	0.140
	Group	6.83	2	179	0.001	0.071

Partial *η*^2^, Partial Eta Squared.

As Table 5 shows, to assess whether participants in the stress management and communication skills groups showed greater reductions in negative emotional states than the control group, three mixed-design ANOVAs were conducted with Time (pre-intervention and post-intervention) as a within-subjects factor and Group (control, stress management, communication) as a between-subjects factor. The dependent variables were FEAR, ANGER, and SADNESS. For all three variables, Mauchly’s Test of Sphericity indicated that the assumption of sphericity was met, with *W* = 1.000 in each case and no significant violations. Therefore, the analyses used the unadjusted degrees of freedom for the interpretation of repeated-measures effects.

**Table 5:** Estimated marginal means for negative emotions by group and time.

Emotion	Group	Time	Mean	SE	95% CI Lower	95% CI Upper
FEAR	Control	Pre	3.247	0.103	3.043	3.451
		Post	3.215	0.088	3.041	3.389
	Stress Mgmt	Pre	3.565	0.071	3.424	3.705
		Post	3.140	0.108	2.927	3.352
	Communication	Pre	3.069	0.096	2.879	3.259
		Post	3.040	0.105	2.832	3.248
ANGER	Control	Pre	3.548	0.079	3.392	3.704
		Post	3.419	0.085	3.252	3.587
	Stress Mgmt	Pre	3.376	0.078	3.222	3.530
		Post	3.376	0.061	3.256	3.497
	Communication	Pre	3.287	0.078	3.133	3.440
		Post	3.374	0.051	3.273	3.475
SADNESS	Control	Pre	3.634	0.062	3.512	3.756
		Post	3.667	0.074	3.521	3.813
	Stress Mgmt	Pre	3.425	0.074	3.280	3.570
		Post	3.360	0.062	3.238	3.481
	Communication	Pre	3.270	0.065	3.142	3.399
		Post	3.707	0.080	3.549	3.866

SE, Standard Error; CI, Confidence Interval.

For FEAR, the analysis revealed a significant main effect of Time and a significant Time by Group interaction, indicating that fear levels generally decreased across the sample and that the magnitude of change differed by group. Estimated marginal means suggested a more substantial reduction in the stress management group compared to the others, although the between-group differences were marginal. For ANGER, there was no significant main effect of Time, suggesting no overall change in anger from pre- to post-intervention. However, a significant Time by Group interaction was detected, indicating differential changes across the groups. Descriptively, the stress management and communication groups showed slight reductions, while the control group’s anger levels remained stable, although no significant between-group effects emerged.

As Table 6 shows, for SADNESS, both the main effect of Time and the time-by-group interaction were significant. *Post hoc* comparisons revealed that sadness levels decreased significantly for participants in the stress management group but increased slightly in the control group, with the communication group showing a moderate reduction. These results support the hypothesis that the intervention groups experienced greater reductions in negative affective states, particularly for SADNESS and, to a lesser extent, FEAR.

**Table 6:** Multivariate and univariate ANOVA results for negative emotions.

Emotion	Effect	*F*	df (Effect)	df (Error)	*p*-value	Partial *η*^2^
FEAR	Time	12.26	1	179	<0.001	0.064
	Time × Group	8.17	2	179	<0.001	0.084
	Group	2.91	2	179	0.057	0.031
ANGER	Time	0.17	1	179	0.682	0.001
	Time × Group	3.22	2	179	0.042	0.035
	Group	1.40	2	179	0.249	0.015
SADNESS	Time	15.50	1	179	<0.001	0.080
	Time × Group	19.69	2	179	<0.001	0.180
	Group	4.30	2	179	0.015	0.046

Partial *η*^2^, Partial Eta Squared.

*Hypothesis 2:* Participants in the experimental groups will report higher satisfaction with social interactions and Work engagement levels post-intervention compared to the control group.

To test Hypothesis 2, two mixed-design ANOVAs were conducted with Time (pre- and post-intervention) as the within-subjects factor and Group (control, social interaction stress management, and effective communication) as the between-subjects factor. The dependent variables were Work engagement and satisfaction with social interactions, as Table 7 shows.

**Table 7:** Estimated marginal means for work engagement and satisfaction by group and time.

Variable	Group	Time	Mean	SE	95% CI Lower	95% CI Upper
Work engagement	Control	Pre	3.129	0.069	2.992	3.267
		Post	3.026	0.059	2.909	3.142
	Stress Mgmt	Pre	2.971	0.061	2.851	3.092
		Post	3.084	0.062	2.962	3.205
	Communication	Pre	2.866	0.059	2.749	2.984
		Post	2.962	0.060	2.843	3.080
Satisfaction	Control	Pre	2.650	0.059	2.533	2.767
		Post	2.610	0.056	2.499	2.721
	Stress Mgmt	Pre	2.660	0.050	2.561	2.759
		Post	2.723	0.070	2.585	2.861
	Communication	Pre	2.684	0.051	2.582	2.786
		Post	2.738	0.062	2.616	2.859

SE, Standard Error; CI, Confidence Interval.

Mauchly’s Test of Sphericity confirmed that the assumption of sphericity was met for both Work engagement and satisfaction, with a *W* value of 1.000 in both cases, indicating no significant violations. Therefore, unadjusted degrees of freedom were used to interpret the within-subject’s effects.

For Work engagement, as Table 8 shows, there was no significant main effect of Time, indicating no overall change in Work engagement from pre- to post-intervention. However, the Time by Group interaction was statistically significant, suggesting that the direction and magnitude of Work engagement change differed among the groups. Post hoc contrasts revealed that the stress management group exhibited a slight increase in Work engagement, whereas the control and communication groups showed minor decreases; however, no significant between-group differences were observed at the post-intervention stage.

**Table 8:** Multivariate and univariate ANOVA results for work engagement and social satisfaction.

Variable	Effect	*F*	df (Effect)	df (Error)	*p*-value	Partial *η*^2^
Work engagement	Time	2.58	1	179	0.110	0.014
	Time × Group	10.08	2	179	< 0.001	0.101
	Group	1.99	2	179	0.139	0.022
Satisfaction	Time	1.51	1	174	0.220	0.009
	Time × Group	2.51	2	174	0.084	0.028
	Group	0.57	2	174	0.569	0.006

Partial *η*^2^, Partial Eta Squared.

For satisfaction with social interactions, neither the main effect of Time nor the interaction between Time and Group reached statistical significance. Descriptively, the control group showed a small decrease in satisfaction, while the stress management and communication groups showed modest increases. These changes, however, were not statistically significant.

*Hypothesis 3:* Improvements in emotional states, satisfaction with social interactions, and Work engagement observed post-intervention will be maintained at the end of the academic year.

As Table 9 shows, to examine whether the improvements in positive emotional states observed post-intervention were maintained at the end of the academic year, a series of repeated-measures ANOVAs were conducted with Time (pre-intervention, post-intervention, and three-month follow-up) as the within-subjects factor and Group (stress management, communication) as the between-subjects factor. The dependent variables were SEEKING, CARING, and PLAYING. For each variable, Mauchly’s Test of Sphericity was conducted. The test revealed that the sphericity assumption was violated for SEEKING (*W* = 0.776, *p* < 0.001), CARING (*W* = 0.946, *p* = 0.040), and PLAYING (*W* = 0.938, *p* = 0.023). Therefore, Greenhouse–Geisser corrections were applied to the degrees of freedom in the relevant analyses.

**Table 9:** Estimated means for positive emotions across three time points by group.

Emotion	Group	Pre	Post	Follow-up
SEEKING	Stress Management	2.46	2.83	2.82
	Communication	2.40	2.77	2.72
CARING	Stress Management	3.68	4.31	4.03
	Communication	3.62	4.04	3.96
PLAYING	Stress Management	3.29	3.78	3.49
	Communication	3.14	3.46	3.40

Values are means based on raw descriptive statistics.

As Table 10 shows, across all three positive emotions, the main effect of Time was significant, indicating that the initial gains observed post-intervention were, on average, maintained through the follow-up period. For SEEKING, although the main effect of Time was significant, the Time × Group interaction was not significant, suggesting similar maintenance patterns across both experimental groups. For CARING, the Time × Group interaction reached significance, indicating that changes over time differed slightly between the groups. A quadratic effect emerged, with a reduction from post-test to follow-up that was more pronounced in the communication group, while the stress management group showed greater stability.

**Table 10:** Repeated-measures ANOVA results for positive emotions (Greenhouse–Geisser corrected).

Emotion	Effect	*F*	df (Effect)	df (Error)	*p*-value	Partial *η*^2^
SEEKING	Time	34.23	1.63	192.79	<0.001	0.225
	Time × Group	0.09	1.63	192.79	0.880	0.001
CARING	Time	81.11	1.90	223.99	<0.001	0.407
	Time × Group	3.98	1.90	223.99	0.022	0.033
PLAYING	Time	29.72	1.88	222.13	<0.001	0.201
	Time × Group	2.55	1.88	222.13	0.084	0.021

Partial *η*^2^, Partial Eta Squared.

For PLAYING, the Time × Group interaction approached significance, indicating a trend toward group differences in how PLAYING levels evolved. Specifically, while both groups showed improvement from pre- to post-intervention, a mild reduction was observed at follow-up, particularly in the communication group. Overall, these findings suggest that gains in positive emotional states following the intervention were largely preserved at the end of the academic year, particularly in the stress management group.

As Table 11 shows, to assess whether reductions in negative emotional states were maintained at the end of the academic year, repeated measures ANOVAs were conducted for ANGER, SADNESS, and FEAR across three time points (pre-intervention, post-intervention, and 3 months later) among the two experimental groups: social interaction stress management and effective communication. For ANGER, the Mauchly’s Test of Sphericity indicated that the assumption of sphericity was not violated, *W* = 0.968, *χ*^2^(2) = 3.792, *p* = 0.150. Therefore, the results assuming sphericity were interpreted. There was a significant main effect of time, *F*(2, 236) = 8.454, *p* < 0.001, *η*^2^ₚ = 0.067, indicating a significant reduction in anger over time. However, the interaction between time and group was not significant, *F*(2, 236) = 0.933, *p* = 0.395, indicating that both experimental groups exhibited similar patterns of anger reduction across the three time points.

**Table 11:** Repeated measures ANOVA for negative emotions (ANGER, SADNESS, and FEAR).

Emotion	Effect	*F*	df (Effect, Error)	*p*-value	Partial *η*^2^
ANGER	Time	8.45	(2, 236)	<0.001	0.067
	Time × Group	0.93	(2, 236)	0.395	0.008
SADNESS	Time	17.74	(2, 228)	<0.001	0.135
	Time × Group	13.43	(2, 228)	<0.001	0.105
FEAR	Time	10.53	(2, 236)	<0.001	0.082
	Time × Group	8.42	(2, 236)	<0.001	0.067

Partial *η*^2^, Partial Eta Squared.

Regarding SADNESS, the Mauchly’s test showed a violation of sphericity, *W* = 0.829, *χ*^2^(2) = 21.206, *p* < 0.001. Therefore, Greenhouse–Geisser corrected values were used. A significant main effect of time was observed, *F*(1.708, 194.687) = 17.735, *p* < 0.001, *η*^2^ₚ = 0.135, reflecting a significant change in sadness levels over time. Crucially, a significant interaction between time and group was found, *F*(1.708, 194.687) = 13.427, *p* < 0.001, *η*^2^ₚ = 0.105. Follow-up contrasts revealed that the effective communication group initially experienced an increase in sadness from pre- to post-intervention, followed by a decline at follow-up. In contrast, the stress management group demonstrated a gradual and consistent decrease.

For FEAR, the Mauchly’s test was violated, *W* = 0.757, *χ*^2^(2) = 32.532, *p* < 0.001, and Greenhouse–Geisser corrections were applied. There was a significant main effect of time, *F*(1.609, 189.902) = 10.534, *p* < 0.001, *η*^2^ₚ = 0.082, indicating a significant overall decrease in fear across the three time points. Importantly, a significant time by group interaction was also observed, *F*(1.609, 189.902) = 8.422, *p* < 0.001, *η*^2^ₚ = 0.067. Simple effect analyses indicated that the social interaction stress management group experienced a more pronounced reduction in fear than the effective communication group, particularly between the pre- and post-intervention periods.

Finally, as Table 12 shows, to assess whether the improvements in Work engagement and social satisfaction observed immediately after the intervention were maintained 3 months later, two repeated-measures ANOVAs were conducted on teacher Work engagement and satisfaction with social interactions. Each analysis included three time points (pre-intervention, post-intervention, and follow-up), with a focus exclusively on the two experimental groups.

**Table 12:** Repeated measures ANOVA for teacher work engagement and social interaction satisfaction (3 time points).

Outcome	Effect	*F*	df (Effect, Error)	*p*-value	Partial *η*^2^
Teacher work engagement	Time	13.58	(2, 236)	<0.001	0.103
	Time × Group	0.06	(2, 236)	0.944	0.000
Social interaction satisfaction	Time	3.10	(2, 214)	0.047	0.028
	Time × Group	1.36	(2, 214)	0.258	0.013

Partial *η*^2^, Partial Eta Squared.

For teachers, work engagement results revealed a significant main effect of time, indicating changes in Work engagement levels over time. However, the interaction between time and group was not significant, suggesting that both experimental groups followed similar patterns in Work engagement changes across the three time points.

Regarding satisfaction with social interactions, there was a statistically significant main effect of time. The interaction effect between time and group was not significant, indicating that the trajectories of change were comparable across the two intervention conditions.

## Discussion

### Hypothesis 1: changes in positive and negative emotions

The findings support Hypothesis 1, indicating that participants in the experimental groups—particularly those in the stress management condition—showed significant increases in primary positive emotional states (SEEKING, CARING, and PLAYING) and significant reductions in negative emotional states (FEAR, ANGER, and SADNESS) relative to the control group. These results align with the principles of the Affective Neuroscience Theory (ANT), which emphasizes the subcortical origins and functional relevance of primary emotional systems in shaping behavior and mental health (Davis and Montag, 2019; Panksepp et al., 2017).

The improvements in positive emotions, especially CARING and PLAYING, reflect findings from brief, structured interventions—such as mindfulness-based strategies and digital tools—that have demonstrated small to moderate increases in positive affect (Prydz et al., 2024; Schürmann-Vengels et al., 2022). The current study contributes to this evidence by demonstrating that classroom-based, peer-supported sessions can also activate SEEKING-like states and enhance participants’ social joy and empathy (Hurling et al., 2017).

The observed reductions in negative emotions, particularly in SADNESS and FEAR, are consistent with interventions emphasizing positive emotion regulation (Byrne and Gustafsson, 2024) and educational programs designed to foster emotional literacy and coping in structured environments (Cook et al., 2020). While anger reductions were less pronounced, the presence of a significant Time × Group interaction suggests that even more reactive or defensive emotional systems may be responsive to targeted intervention over time.

These findings support the broader theoretical proposition that brief affective interventions, when informed by neuroscience and grounded in practice, can be linked to meaningful modulation in the intensity of core emotional traits, even in adult populations. They confirm the potential of structured programs to support emotional regulation, aligning with frameworks like the process model of emotion regulation (Quoidbach et al., 2015) and extending evidence for plasticity within the SEEKING, CARING, and PLAYING circuits.

### Hypothesis 2: work engagement and social interaction satisfaction

Hypothesis 2 predicted that participants in the experimental groups would report greater improvements in work engagement and satisfaction with social interactions compared to the control group following the intervention. The results partially supported this prediction. While the stress management group showed a modest but statistically significant increase in work engagement, no statistically significant between-group differences were observed for satisfaction with social interactions, although descriptive trends indicated improvement among intervention participants.

The engagement findings align with broader evidence linking emotional regulation to psychological capital, including hope, optimism, and resilience, which are known to mediate engagement outcomes (Ma, 2023). Likewise, practices such as mindfulness, when integrated into daily life, have been shown to foster positive affect and intrinsic motivation, which in turn promote professional fulfillment (Tao, 2022).

Although the changes in social satisfaction did not reach significance, the descriptive increase in both intervention groups is worth noting. Programs that enhance emotional intelligence and empathy—like the communication training used in this study—have been linked to improved interpersonal relationships in school environments (Chiracu et al., 2024; Kiptiony, 2024). Moreover, educational systems that promote social–emotional learning tend to cultivate a more positive climate for both students and teachers (Chen and Yu, 2022).

The observed engagement improvements are also consistent with theories emphasizing autonomy support and need satisfaction (Zhang et al., 2021). Activities that allowed for self-reflection, expression of empathy, and stress coping may have indirectly contributed to a sense of agency, contributing to motivational gains. Furthermore, the importance of the positivity ratio in sustaining wellbeing and engagement (Rusu and Colomeischi, 2020) may help explain why emotion-centered interventions translated into modest but observable changes in workplace commitment. Taken together, these findings suggest that while brief affective interventions may be sufficient to influence motivational outcomes such as engagement, more intensive or prolonged relational components may be required to produce robust changes in perceived social satisfaction.

### Hypothesis 3: maintenance of emotional and behavioral gains

Hypothesis 3 addressed whether the emotional and occupational improvements observed post-intervention would be maintained at the end of the academic year. The data support this hypothesis. Both positive and negative emotional changes were largely sustained at follow-up, with particularly strong stability in the stress management group. Work engagement and social satisfaction also remained consistent across the three time points.

These outcomes echo a growing body of literature suggesting that interventions targeting affective regulation and resilience can yield lasting change. For example, Wang Y. et al. (2025) demonstrated that emotion regulation training among education workers produced sustained reductions in stress and improved job performance up to 6 months later. Similarly, Dolcos et al. (2021) found that such training produced both psychological and neural changes associated with long-term wellbeing.

In the current study, the consistent improvement in CARING and PLAYING, along with the stabilization of SEEKING, reflects the persistence of positive affective traits following training. These results align with prior findings from interventions that have shown long-term benefits in mood, self-esteem, and emotion regulation, supported by shifts in underlying neurocognitive processes (Mrazek et al., 2016; Schweizer et al., 2013). Although anger showed less change, the pattern of sustained reduction across both intervention groups reinforces the notion that even less malleable emotional traits may exhibit gradual change with sufficient time and practice. The durability of work engagement and social satisfaction is also notable, suggesting that emotional changes are generalized to occupational and interpersonal contexts.

From a conceptual perspective, the present findings are consistent with a theory-driven interpretation in which changes in primary emotional systems serve as proximal affective shifts that may support downstream motivational and social outcomes. Although no formal mediation analyses were conducted, ANT provides a coherent framework for understanding how the modulation of SEEKING, CARING, and PLAYING, alongside reductions in FEAR and SADNESS, may create affective conditions conducive to greater engagement and relational wellbeing. This interpretation is offered as a conceptual model rather than as evidence of a specific causal pathway.

However, consistent with broader findings in applied psychology (Ambrose et al., 1998; Page and Vella-Brodrick, 2013), the possibility of diminishing returns over time underscores the need for reinforcement. Programs that include follow-up sessions, reflective activities, or community-based support are more likely to sustain benefits. Multi-component curricula, such as the PROMEHS program, which integrates emotional education with resilience-building strategies, have demonstrated lasting improvements in teacher wellbeing and social functioning (Cavioni et al., 2023), suggesting a viable path forward for institutionalizing such approaches.

In summary, the present findings affirm that brief interventions targeting affective systems at their neuropsychological roots can be associated with durable benefits across emotional, motivational, and social domains. However, the sustainability of these gains may depend on factors such as intervention reinforcement, individual differences in emotional plasticity, and environmental support. Designing interventions with built-in continuity strategies—such as follow-up prompts, community building, or institutional integration—may enhance the long-term effectiveness of programs aimed at strengthening teachers’ emotional and interpersonal wellbeing. This aligns with recent multi-component initiatives, such as the PROMEHS program, which emphasizes emotional education, social competence, and resilience-building in teachers. The program demonstrated sustained improvements in mental health and professional adjustment over time, indicating that integrated, theory-driven curricula can foster both immediate and long-term benefits when emotional development is prioritized in educational contexts (Cavioni et al., 2024).

### Limitations and suggestions for future research

Despite the promising findings of this study, several limitations should be acknowledged that may impact the interpretation and generalizability of the results.

All outcome variables, including emotional traits, work engagement, and social interaction satisfaction, were assessed through self-report questionnaires. While validated instruments such as the ANPS and the C-UWES-3 were used, self-report data can be subject to social desirability bias, recall errors, and response tendencies, particularly in intervention settings. Future studies may benefit from integrating multi-method approaches, including behavioral tasks, teacher evaluations, or physiological measures, to corroborate subjective outcomes and capture broader dimensions of emotional and occupational functioning (Arbel et al., 2024).

In addition, the interpretation of the findings is guided by theory-driven conceptual assumptions derived from Affective Neuroscience Theory. While these assumptions provide a coherent framework for understanding patterns of affective change, they do not constitute formal tests of causal or mediational processes. Consequently, the conclusions should be understood as theoretically informed interpretations rather than as evidence of specific underlying mechanisms.

The follow-up assessment was conducted 3 months after the intervention, which, although informative, provides only a short-term view of the intervention’s sustainability. Longer-term follow-up periods—6 months to 1 year—would be valuable in establishing the durability of the observed improvements and determining whether booster sessions or reinforcement strategies are necessary to maintain gains over time. This is particularly important for complex constructs such as work engagement and interpersonal satisfaction, which may fluctuate in response to institutional demands and life events.

Although the interventions aimed to improve outcomes relevant to professional and social life, no objective data (e.g., job performance evaluations, absenteeism rates, or peer interaction quality) were collected to substantiate improvements beyond self-perceived change. Including such indicators in future research could strengthen conclusions regarding real-world impact and facilitate organizational implementation of similar programs.

The study was conducted in Chinese secondary schools, which may limit generalizability to other cultural or educational contexts. Emotional norms, interpersonal dynamics, and workplace structures vary across countries and sectors. While the use of culturally validated instruments (e.g., the C-UWES-3) enhances internal validity, replication in diverse cultural contexts is essential for evaluating cross-cultural applicability. Additionally, although participants were drawn from multiple districts, the lack of school-level controls means that institutional variables (e.g., leadership style, school climate) were not accounted for, which could potentially influence the efficacy of the intervention (Sott and Bender, 2025).

Beyond age, gender, and marital status, no additional demographic or contextual variables (e.g., teaching experience, subject area, workload, or school type) were collected. Such factors may moderate intervention responsiveness and could have provided deeper insights into the individual and environmental determinants of change. Collecting a broader set of contextual variables would allow for subgroup analyses and a more nuanced understanding of how personal and professional characteristics influence outcomes. The control group received written materials describing the interventions but did not participate in any structured training activities. Although this approach was ethically justified and accompanied by a debriefing session, it does not control for placebo effects or group contact, which may inflate observed group differences. Future studies could consider active control conditions, such as alternative training pieces or matched attention sessions, to enhance the robustness of group comparisons (Haynos et al., 2023).

Although participant retention was high overall, follow-up data were only collected for the two intervention groups. This limits the ability to compare long-term changes with the control group and may introduce selective attrition bias if participants who remained engaged differed systematically from those who dropped out. Future research should aim for complete longitudinal tracking across all groups to minimize bias and support stronger causal inferences.

### Future directions and practical implications

The results of this study offer meaningful directions for future research and inform practical considerations for educational systems seeking to support teacher wellbeing through affective interventions.

One key direction for future research involves extending the follow-up period beyond 3 months to examine the long-term durability of intervention effects. While the current study demonstrated sustained improvements in emotional states and work engagement at the end of the academic year, it remains unclear whether these gains persist across subsequent school terms or academic cycles. Prior research has demonstrated that the positive effects of workplace interventions on wellbeing can diminish over time without ongoing support (Carl et al., 2018). Longitudinal designs with assessments 6 or 12 months post-intervention would help determine the degree of decay or consolidation of emotional gains, as earlier studies on other context showed (Finkel et al., 2013).

Future studies should also employ multi-method approaches that include behavioral indicators or third-party evaluations. The exclusive reliance on self-report data in the present study, while common in affective neuroscience research, may limit insight into how trait-level emotional changes influence observable classroom behavior or relational dynamics. Incorporating observational assessments, peer-reports, or physiological measures (e.g., cortisol, heart rate variability) would provide converging evidence on the effectiveness of affective interventions (Matt et al., 2024).

Moreover, future research should investigate potential moderators of intervention effectiveness, including baseline emotional profiles, years of teaching experience, workload, and school-level factors such as leadership support and organizational climate. Individual differences in emotional plasticity or stress vulnerability may shape how teachers respond to emotion-focused programs (Mrazek et al., 2016). Identifying such moderators could inform more targeted and adaptive implementations.

Another promising avenue involves integrating emotion-based approaches with complementary psychological frameworks, including self-determination theory, positive psychology, and the Job Demands-Resources (JD-R) model. Research has shown that emotional regulation strategies enhance psychological capital, encompassing hope, optimism, self-efficacy, and resilience, which in turn mediates the relationship between emotions and engagement (Wang K. et al., 2025). Future studies may examine how affective and motivational pathways jointly contribute to teacher wellbeing and institutional functioning.

Cultural context also warrants greater attention. Although the present study employed validated Chinese-language instruments and culturally adapted procedures, the generalizability of ANT-based interventions across diverse educational systems remains an open question. Programs such as PROMEHS, which integrate social–emotional learning and wellbeing strategies across European contexts, demonstrate the potential of culturally flexible curricula to support teacher mental health (Wang et al., 2020). Cross-national replications and comparative studies would provide insight into how cultural values and educational structures shape affective systems (Wang et al., 2021).

From a practical standpoint, the findings suggest that brief, neuroscience-informed interventions can be feasibly implemented within school settings to enhance emotional wellbeing and professional engagement. The four-week stress management and communication programs employed in this study yielded measurable improvements without requiring extensive resources or disrupting teaching schedules. This aligns with other research supporting the cost-effectiveness of short interventions in occupational health (Wang et al., 2024).

Furthermore, the evidence that gains were maintained over time supports the integration of such programs into teacher professional development. Interventions targeting core emotional systems—particularly those enhancing SEEKING, CARING, and PLAYING—may function as protective resources against burnout and relational fatigue, especially in high-stress environments. The results also reinforce the importance of institutional support in maintaining wellbeing; schools that embed emotional skills training within broader professional development frameworks are more likely to sustain positive outcomes (Proyer et al., 2020).

Finally, findings suggest that emotional interventions should not be viewed in isolation but rather as components of a systemic approach to teacher wellbeing. Affective training can complement efforts to enhance workplace climate, leadership support, and autonomy. For example, research by Zhang et al. (2021) demonstrates that teachers’ perceived autonomy support contributes to engagement and social satisfaction via intrinsic motivation. Integrating emotion-based programs with autonomy-promoting structures may yield synergistic effects.

In conclusion, brief affective interventions grounded in Affective Neuroscience Theory offer a promising, scalable method for promoting emotional resilience and professional engagement among educators. Future research should continue to refine these models, expand their scope, and test their sustainability across contexts. At the same time, educational systems should consider institutionalizing such programs as part of a comprehensive strategy to promote emotional competence, psychological wellbeing, and relational health within the teaching profession.

## Conclusion

This study contributes to the growing body of research demonstrating that brief, neuroscience-informed interventions may be associated with improvements in emotional wellbeing, improve satisfaction with social interactions, and foster professional engagement among secondary school teachers. Grounded in Affective Neuroscience Theory (ANT), the intervention programs were designed to target core emotional systems—SEEKING, CARING, and PLAYING—while also regulating negative emotional traits such as FEAR, ANGER, and SADNESS. The results confirm that such interventions can be linked to immediate improvements in emotional and occupational functioning and that these changes may be sustained over time.

Participants in the stress management and communication skills training groups exhibited greater increases in positive emotions and reductions in negative affect compared to a control group, with particularly robust effects observed for CARING, PLAYING, and SADNESS. Improvements in work engagement and social satisfaction were also observed, especially in the stress management group, although these effects were more modest, supporting the premise that emotional interventions can generalize to broader domains of professional and relational life. Importantly, these changes were largely sustained 3 months after the interventions, suggesting that even brief programs may initiate enduring transformation in core emotional traits when grounded in a solid neuropsychological framework.

The findings underscore the importance of integrating affective neuroscience into the design of teacher development programs, offering practical implications for education policy and institutional practice. By targeting foundational emotional systems, such approaches may support not only teachers’ mental health but also their capacity for empathy, resilience, and relational connection in the school environment. As educational systems worldwide grapple with teacher stress, burnout, and disengagement, the present study highlights the potential value of brief, theory-driven interventions as feasible and scalable components of broader wellbeing strategies in education.

Future research should build upon these insights by exploring long-term outcomes, contextual moderators, and the integration of emotional training with systemic organizational support. Ultimately, this work reinforces the notion that emotional development is not peripheral but central to professional competence and satisfaction and that cultivating positive affective systems may provide an important foundation for fostering sustainable and supportive educational communities.

## Data Availability

The raw data supporting the conclusions of this article will be made available by the authors without undue reservation.

## Appendix A: Intervention programs

Both intervention programs were designed and delivered by the research team in collaboration with trained facilitators. The interventions took place in a dedicated space within the host institution’s facilities. Each program consisted of four weekly sessions, delivered over a one-month period, with each session lasting approximately 2 h. Sessions were conducted in small groups and followed a structured format, including psychoeducation, experiential activities, and group reflection.

### Communication skills program

The Communication Skills Program aimed to enhance participants’ competence in verbal and nonverbal communication, empathy, and active listening during social interactions. The intervention was grounded in theories of interpersonal communication and principles of experiential learning. The specific content of each session is outlined below:

- Session 1—Foundations of Effective Communication:
- Introduction to the fundamental principles of interpersonal communication, including awareness of verbal and nonverbal signals, and exercises to identify barriers and facilitators of communication.
- Session 2—Active Listening and Empathic Understanding:
- Training in active listening techniques, paraphrasing, and emotional validation; role-plays to practice responding empathetically in everyday situations.
- Session 3—Expressing Needs and Emotions:
- Focused on assertiveness and self-expression, with activities to practice “I-statements” and constructively express personal thoughts and feelings in social contexts.
- Session 4—Integrating Communication Strategies:
- Simulation of real-life social scenarios through role-play and group feedback, with a review of key skills and planning for their application in daily interpersonal situations.

### Social interaction stress management program

The Stress Management Program was designed to equip participants with tools to manage emotional responses and mitigate stress during social interactions. The intervention integrated principles from mindfulness-based interventions and cognitive-behavioral approaches. The content of each session was as follows:

- Session 1—Understanding Stress in Social Contexts:
- Psychoeducation on the physiological and psychological components of stress, including the identification of individual stress triggers in social environments and an introduction to mindful awareness.
- Session 2—Breathing and Attention Regulation Techniques:
- Practice of diaphragmatic breathing and focused attention, along with mindfulness-based exercises, to increase present-moment awareness and reduce automatic stress responses.
- Session 3—Cognitive Restructuring of Stressful Social Thoughts:
- Identification of maladaptive thoughts and beliefs, training in reframing and flexible thinking, and practice through guided reflection and peer exchange.
- Session 4—Coping Strategies and Real-Life Application:
- Development of a personalized coping plan; discussion of challenges in implementing strategies; rehearsal through role-play and feedback.

Both programs were designed to be psychoeducational and experiential, emphasizing the acquisition of skills, practice, and their transfer to real-life social and academic settings.
