# Supplementary material for: Social interactions and affective neuroscience personality traits among Chinese educators: a randomized intervention study on wellbeing
Source: Front Psychol. 2026 Feb 20;17:1712521. doi: 10.3389/fpsyg.2026.1712521 (PMC12963315; doi:10.3389/fpsyg.2026.1712521)
Supplement: Supplementary file 1 [file Table_1.DOCX]

**Table S1. Pearson’s matrix correlation** *(N=182/112)*

| **Variables** | **1.** | **2.** | **3.** | **4.** | **5.** | **6.** | **7.** | **8.** | **9.** | **10.** | **11.** | **12.** | **13.** | **14.** | **15.** | **16.** | **17.** | **18.** | **19.** | **20.** | **21.** | **22.** | **23.** |
| --- | --- | --- | --- | --- | --- | --- | --- | --- | --- | --- | --- | --- | --- | --- | --- | --- | --- | --- | --- | --- | --- | --- | --- |
| 1. Seeking | -- |  |  |  |  |  |  |  |  |  |  |  |  |  |  |  |  |  |  |  |  |  |  |
| 2. Caring | .043 | -- |  |  |  |  |  |  |  |  |  |  |  |  |  |  |  |  |  |  |  |  |  |
| 3. Playing | .399^**^ | .424^**^ | -- |  |  |  |  |  |  |  |  |  |  |  |  |  |  |  |  |  |  |  |  |
| 4. Seeking Post | .319^**^ | .039 | .299^**^ | -- |  |  |  |  |  |  |  |  |  |  |  |  |  |  |  |  |  |  |  |
| 5. Caring Post | .232^**^ | .500^**^ | .589^**^ | .276^**^ | -- |  |  |  |  |  |  |  |  |  |  |  |  |  |  |  |  |  |  |
| 6. Playing Post | .198^**^ | .401^**^ | .482^**^ | .150^*^ | .693^**^ | -- |  |  |  |  |  |  |  |  |  |  |  |  |  |  |  |  |  |
| 7. Seeking End Course | .445^**^ | .170 | .480^**^ | .806^**^ | .440^**^ | .180^*^ | -- |  |  |  |  |  |  |  |  |  |  |  |  |  |  |  |  |
| 8. Caring End Course | .002 | .649^**^ | .571^**^ | .317^**^ | .674^**^ | .424^**^ | .584^**^ | -- |  |  |  |  |  |  |  |  |  |  |  |  |  |  |  |
| 9. Playing End Course | .240^**^ | .078 | .544^**^ | .608^**^ | .218^*^ | .423^**^ | .727^**^ | .498^**^ | -- |  |  |  |  |  |  |  |  |  |  |  |  |  |  |
| 10. Anger | -.164^*^ | -.156^*^ | -.232^**^ | -.352^**^ | -.107 | -.153^*^ | -.510^**^ | -.229^*^ | -.526^**^ | -- |  |  |  |  |  |  |  |  |  |  |  |  |  |
| 11. Sadness | -.139 | -.304^**^ | -.284^**^ | -.378^**^ | -.330^**^ | -.076 | -.262^**^ | -.289^**^ | .048 | .373^**^ | -- |  |  |  |  |  |  |  |  |  |  |  |  |
| 12. Fear | -.135 | -.015 | .091 | .023 | .100 | -.109 | -.108 | -.046 | -.113 | .215^**^ | .112 | -- |  |  |  |  |  |  |  |  |  |  |  |
| 13. Anger Post | -.178^*^ | -.149^*^ | -.164^*^ | -.357^**^ | -.338^**^ | -.160^*^ | -.567^**^ | -.365^**^ | -.374^**^ | .662^**^ | .343^**^ | .061 | -- |  |  |  |  |  |  |  |  |  |  |
| 14. Sadness Post | -.044 | -.273^**^ | -.213^**^ | -.323^**^ | -.318^**^ | -.114 | -.276^**^ | -.311^**^ | .018 | .105 | .597^**^ | .030 | .332^**^ | -- |  |  |  |  |  |  |  |  |  |
| 15. Fear Post | -.132 | -.080 | -.223^**^ | -.359^**^ | -.149^*^ | -.224^**^ | -.485^**^ | -.358^**^ | -.371^**^ | .224^**^ | .322^**^ | .636^**^ | .130 | .411^**^ | -- |  |  |  |  |  |  |  |  |
| 16. Anger End Course | -.225^*^ | -.481^**^ | -.354^**^ | -.312^**^ | -.472^**^ | -.151 | -.731^**^ | -.609^**^ | -.451^**^ | .518^**^ | .114 | .103 | .473^**^ | .045 | .280^**^ | -- |  |  |  |  |  |  |  |
| 17. Sadness End Course | .092 | -.438^**^ | -.524^**^ | -.473^**^ | -.343^**^ | -.245^**^ | -.415^**^ | -.525^**^ | -.389^**^ | .203^*^ | .350^**^ | .017 | .141 | .465^**^ | .620^**^ | .409^**^ | -- |  |  |  |  |  |  |
| 18. Fear End Course | -.224^*^ | .010 | .007 | -.416^**^ | .186^*^ | -.034 | -.393^**^ | -.281^**^ | -.260^**^ | .097 | .224^*^ | .634^**^ | -.126 | .328^**^ | .865^**^ | .141 | .323^**^ | -- |  |  |  |  |  |
| 19. Work engagement | .312^**^ | .173^*^ | .358^**^ | .397^**^ | .055 | .094 | .438^**^ | .035 | .456^**^ | -.210^**^ | -.086 | -.068 | -.098 | -.033 | -.138 | -.241^**^ | -.341^**^ | -.074 | -- |  |  |  |  |
| 20. Work engagement Post | .194^**^ | .315^**^ | .401^**^ | .577^**^ | .188^*^ | .188^*^ | .719^**^ | .283^**^ | .579^**^ | -.253^**^ | -.279^**^ | -.094 | -.177^*^ | -.323^**^ | -.403^**^ | -.406^**^ | -.612^**^ | -.424^**^ | .796^**^ | -- |  |  |  |
| 21. Work engagement End Course | .140 | -.071 | .025 | .565^**^ | -.065 | .257^**^ | .446^**^ | .177 | .544^**^ | -.231^*^ | .322^**^ | -.161 | -.258^**^ | .110 | -.291^**^ | -.151 | -.271^**^ | -.263^**^ | .679^**^ | .611^**^ | -- |  |  |
| 22. Satisfaction Social Interaction | .366^**^ | .199^**^ | .318^**^ | .427^**^ | .405^**^ | .434^**^ | .563^**^ | .617^**^ | .536^**^ | -.177^*^ | -.239^**^ | -.411^**^ | -.188^*^ | -.227^**^ | -.549^**^ | -.231^*^ | -.323^**^ | -.527^**^ | .237^**^ | .263^**^ | .529^**^ | -- |  |
| 23. Satisfaction Social Interaction Post | .256^**^ | .337^**^ | .392^**^ | .531^**^ | .494^**^ | .505^**^ | .563^**^ | .694^**^ | .536^**^ | -.244^**^ | -.375^**^ | -.259^**^ | -.364^**^ | -.398^**^ | -.567^**^ | -.250^**^ | -.463^**^ | -.579^**^ | .065 | .282^**^ | .337^**^ | .817^**^ | -- |
| 24. Satisfaction Social Interaction End Course | .276^**^ | .255^**^ | .307^**^ | .550^**^ | .285^**^ | .413^**^ | .726^**^ | .723^**^ | .675^**^ | -.329^**^ | -.178 | -.426^**^ | -.279^**^ | -.319^**^ | -.641^**^ | -.459^**^ | -.414^**^ | -.719^**^ | .139 | .478^**^ | .439^**^ | .817^**^ | .800^**^ |

*Note*: ** *p<*.001; **p<*.05.
